# Post-operative early enteral nutrition intolerance in elderly patients undergoing laparoscopic gastric cancer surgery: current status and nursing strategies

**DOI:** 10.3389/fnut.2026.1777356

**Published:** 2026-05-18

**Authors:** Suqing Chen, Wenjuan Xu, Suqin Xu, Huimin Yao

**Affiliations:** 1 Department of Gastrointestinal Center, Shanxi Bethune Hospital, Shanxi Academy of Medical Sciences, Tongji Shanxi Hospital, Third Hospital of Shanxi Medical University, Taiyuan, China; 2 Department of Respiratory Medicine, Tongji Hospital Affiliated to Tongji Medical College of Huazhong University of Science and Technology, Wuhan, Hubei, China

**Keywords:** elderly, enteral nutrition, gastric cancer, intolerance, nursing, surgery

## Abstract

**Background:**

Early enteral nutrition (EEN) is a core component of perioperative management for elderly patients undergoing laparoscopic gastric cancer surgery, but nutritional intolerance is a common clinical problem that impairs the effectiveness of nutritional support. This study aimed to evaluate the influencing factors of post-operative EEN intolerance and constructing a predictive model for clinical nursing management.

**Methods:**

A retrospective case-control study was conducted, elderly patients undergoing laparoscopic gastric cancer surgery were included. Correlation analysis and Logistic regression analysis were performed to screen independent risk factors of EEN intolerance. A predictive model was constructed based on the independent risk factors, and the receiver operating characteristic (ROC) curve was used to evaluate its predictive efficacy.

**Results:**

A total of 346 elderly patients undergoing laparoscopic gastric cancer surgery were included, of whom 194 (56.1%) developed post-operative EEN intolerance. Spearman correlation analysis identified significant positive correlations between EEN intolerance and age, TNM stage, diabetes mellitus, surgical scope, and intraoperative blood loss (all *P* < 0.001). Multivariate logistic regression analysis revealed that age ≥75 years, TNM stage III–IV comorbid diabetes mellitus, radical resection, and intraoperative blood loss ≥250 ml were independent risk factors for EEN intolerance (all *P* < 0.001). A predictive model incorporating these five factors demonstrated good discriminatory ability, with an area under the curve of 0.856. At the optimal cut-off value of 6.5 points, the model achieved a Youden index of 0.645, with a sensitivity of 0.756 and a specificity of 0.889.

**Conclusion:**

Post-operative EEN intolerance occurs frequently in elderly patients undergoing laparoscopic gastric cancer surgery and is associated with multiple independent risk factors. The constructed predictive model demonstrated good predictive efficacy in this cohort. Clinical application of targeted perioperative management strategies based on these risk factors may help reduce the incidence of EEN intolerance and improve nutritional outcomes in this vulnerable population.

## Introduction

With the acceleration of the global aging population, the proportion of elderly patients with gastric cancer has been on the rise year by year, making them a key population of interest in the field of gastric cancer diagnosis and treatment (1, 2). As a common malignant tumor of the digestive system, surgical resection remains the preferred radical treatment option in clinical practice (3). In recent years, laparoscopic technology has been widely applied in surgeries for elderly gastric cancer patients due to its advantages of minimal invasiveness, rapid post-operative recovery, and low incidence of complications, which has significantly improved the surgical tolerance and short-term post-operative prognosis of elderly patients (4–6). However, elderly patients are generally characterized by physiological function decline, multiple comorbidities, and insufficient nutritional reserves. After surgery, the body is in a state of high stress and high catabolism. As a core component of perioperative management, nutritional support directly affects wound healing, immune function recovery, and long-term quality of life in patients. Among various nutritional support modalities, early enteral nutrition (EEN) has been recommended as the first-line option for post-operative nutritional support in gastric cancer patients by guidelines (7, 8), as it conforms better to the physiological rules of digestion and absorption, preserves the integrity of the intestinal mucosal barrier, and reduces the risk of complications such as infections.

Despite the established benefits of EEN in the post-operative management of elderly patients undergoing laparoscopic gastric cancer surgery, EEN intolerance frequently occurs in clinical practice and remains a major barrier to effective nutritional support (9, 10). EEN intolerance typically manifests as gastrointestinal symptoms including abdominal distension, diarrhea, nausea, and vomiting. These symptoms not only compromise nutrient delivery but may also precipitate adverse outcomes such as dehydration and electrolyte imbalances (11). In severe cases, discontinuation of nutritional support becomes necessary, potentially prolonging hospital stay and increasing healthcare burden (12). Elderly patients are particularly susceptible to EEN intolerance due to age-related physiological changes—including diminished intestinal peristalsis, reduced digestive enzyme secretion, and impaired stress response—compounded by surgical trauma-induced gastrointestinal dysmotility and altered digestive tract anatomy (13–15). Consequently, the incidence and clinical impact of EEN intolerance are more pronounced in this population compared with younger counterparts.

Despite its clinical significance, systematic research on post-operative EEN intolerance in elderly patients undergoing laparoscopic gastric cancer surgery remains limited. A deeper understanding of its prevalence and underlying risk factors is urgently needed to inform targeted nursing interventions. Therefore, this study aimed to investigate the incidence and influencing factors of post-operative EEN intolerance in this specific population, with the goal of providing evidence to optimize perioperative nursing management strategies.

## Methods

### Study design

A retrospective case-control study design was adopted in this research.

### Ethical approval

This study protocol was reviewed and approved by the Medical Ethics Committee of Shanxi Bethune Hospital (Ethics Approval No.: SBQLL-2025–371). Given that this is a retrospective observational study involving the analysis of existing de-identified clinical data, the requirement for obtaining written informed consent from individual participants was waived by the Ethics Committee, in strict compliance with the Declaration of Helsinki and relevant national medical ethical guidelines. All data were processed and analyzed anonymously to protect the privacy and rights of the participants, and the study was conducted without any interference with the participants‘ clinical management or rights and interests.

#### Sample size calculation

Referring to the incidence of early post-operative enteral nutrition intolerance (approximately 50%) in elderly gastric cancer patients reported in previous similar studies (16, 17), the minimum sample size was estimated using the sample size formula for case-control studies: $n=\frac{{\left[Z_{\alpha }/2\sqrt{2P\left(1-P\right)}+Z_{\beta }\sqrt{P_{0}\left(1-P_{0}\right)+P_{1}\left(1-P_{1}\right)}\right]}^{2}}{{\left(P_{1}-P_{0}\right)}^{2}}$. where P_0_ denotes the exposure rate of the control group and P_1_ denotes the exposure rate of the case group. Based on pilot study results, the difference between (P_1_) and P_0_ (P_1_-P_0_) was set at 0.15. The calculated minimum required sample size was 220 cases. In this study, a total of 346 eligible patients were actually enrolled, including 194 cases in the intolerance group and 152 cases in the tolerance group, which met the statistical test requirements of the study.

### Study population

#### Inclusion criteria

(1) Aged ≥ 65 years; (2) Confirmed as gastric cancer by histopathological examination; (3) Underwent laparoscopic radical or palliative gastrectomy; (4) Initiated early enteral nutrition support within 24–72 h after surgery; (5) Complete clinical data, including demographic characteristics, pre-operative comorbidities, surgery-related indicators, nutrition support-related information, and post-operative recovery status.

#### Exclusion criteria

(1) Severe pre-operative gastrointestinal dysfunction (e.g., intestinal obstruction, intestinal perforation); (2) Severe pre-operative malnutrition (serum albumin < 25 g/L) or metabolic diseases (e.g., severe hyperthyroidism, diabetic ketoacidosis); (3) Patients who developed severe post-operative complications (e.g., anastomotic leakage, massive hemorrhage) requiring emergency reoperation; (4) Cognitive impairment or mental illness that prevented cooperation with the study; (5) Incomplete clinical data that affected statistical analysis of the research data.

### Data collection

A retrospective data collection method was employed, and a unified Data Collection Form for Early Post-operative Enteral Nutrition in Elderly Patients Undergoing Laparoscopic Gastric Cancer Surgery was developed. Two systematically trained researchers independently extracted the clinical data of the participants. After extraction, cross-verification was performed. Any discrepancies were resolved through discussion or consultation with senior physicians. The main collected contents included: (1) Demographic data: age, gender, body mass index (BMI); (2) Pre-operative comorbidities: hypertension, diabetes mellitus, pulmonary infection, etc.; (3) Tumor-related indicators: TNM stage; (4) Surgery and anesthesia-related indicators: surgical scope (limited resection/radical resection), operation time, intraoperative blood loss, anesthesia time; (5) Nutrition support-related indicators: nutrition support route (nasointestinal tube/nasogastric tube), initiation time of early post-operative enteral nutrition; (6) Tolerance to early post-operative enteral nutrition: definition of EEN intolerance (18–20): for elderly patients undergoing laparoscopic gastric cancer surgery who received EEN support within 24–72 h after operation, EEN intolerance was defined as the occurrence of one or more gastrointestinal adverse symptoms (including abdominal distension, diarrhea, nausea, vomiting) during the EEN support period, which were confirmed by clinical evaluation to be closely related to enteral nutrition administration and could not be attributed to other causes (such as post-operative complications, drug side effects, or primary gastrointestinal diseases). Among them, abdominal distension was defined as subjective abdominal fullness with or without abdominal circumference increase ≥3 cm compared with the baseline; diarrhea was defined as ≥3 loose or watery stools per day with a total volume >200 ml; nausea was defined as a subjective feeling of wanting to vomit; vomiting was defined as the involuntary expulsion of gastric contents through the mouth. In cases where the above symptoms were mild and did not require adjustment of EEN intensity (rate or dosage), or moderate to severe symptoms that necessitated reduction, suspension, or termination of EEN support, the patients were all classified into the EEN intolerance group. Patients who did not experience any of the above gastrointestinal symptoms during the entire EEN support period were classified into the EEN tolerance group. Presence or absence of intolerant symptoms such as abdominal distension, diarrhea, nausea, vomiting, as well as the onset time and severity of these symptoms.

### Statistical analysis

All statistical analyses were performed using SPSS version 26.0 (IBM Corp., Armonk, NY, USA). Continuous variables were first tested for normality using the Kolmogorov–Smirnov test and for homogeneity of variance using Levene‘s test. Normally distributed continuous data were presented as mean ± standard deviation, and comparisons between two groups were conducted using the independent samples *t*-test. Categorical variables were expressed as frequencies and percentages, and group comparisons were performed using the chi-square test or Fisher‘s exact test, as appropriate. To explore potential associations between clinical factors and post-operative EEN intolerance, Spearman correlation analysis was employed as an exploratory tool, given the ordinal or categorical nature of several variables (e.g., TNM stage, surgical scope). Variables showing statistical significance in univariate analyses or correlation analyses were subsequently entered into a multivariate binary logistic regression model to identify independent risk factors for EEN intolerance. Prior to modeling, multicollinearity among candidate variables was assessed using the variance inflation factor (VIF), with a threshold of VIF < 5 indicating no significant multicollinearity. All VIF values were below 3, confirming the absence of multicollinearity concerns. For the logistic regression analysis, continuous variables (age, intraoperative blood loss) were dichotomized using clinically established cut-off values (e.g., age ≥75 years reflects geriatric vulnerability; blood loss ≥250 ml is a commonly used threshold in surgical risk stratification) based on prior literature and clinical relevance. Although dichotomization may result in some loss of information, this approach was chosen to enhance clinical interpretability and facilitate bedside application. Variables were entered into the regression model using a forward stepwise selection method based on likelihood ratio tests. Results were reported as odds ratios (OR) with 95% confidence intervals (CI). A simplified predictive risk score was subsequently developed based on the independent risk factors identified in the multivariate analysis. For clinical practicality, each risk factor was assigned equal weight (2 points), yielding a total score ranging from 0 to 10. The discriminatory ability of the model was evaluated using receiver operating characteristic (ROC) curve analysis, and the area under the curve (AUC) was calculated. The optimal cut-off value was determined by maximizing the Youden index (sensitivity + specificity – 1). All statistical tests were two-tailed, and a *P*-value < 0.05 was considered statistically significant.

## Results

A total of 346 elderly patients undergoing laparoscopic gastric cancer surgery were enrolled in this study, comprising 194 patients (56.1%) in the EEN intolerance group and 152 patients (43.9%) in the tolerance group. Among the 194 patients classified as intolerant, 58 (29.9%) required reduction, temporary suspension, or permanent termination of EEN due to moderate to severe gastrointestinal symptoms, while the remaining 136 (70.1%) experienced mild symptoms that did not necessitate adjustment of nutritional support.

Comparison of baseline data between the two groups revealed that there were statistically significant differences in age, BMI, TNM stage distribution, prevalence of diabetes mellitus, surgical scope, operation time, intraoperative blood loss, anesthesia time, and initiation time of early post-operative enteral nutrition (all *P* < 0.05). Specifically, patients in the intolerance group were older, had lower BMI, presented a higher proportion of TNM stage III–IV and showed a higher prevalence of diabetes mellitus. They were also more likely to undergo radical resection, with longer operation and anesthesia time, greater intraoperative blood loss, and later initiation of enteral nutrition. No statistically significant differences were observed between the two groups in gender distribution, prevalence of hypertension, and route of nutritional support (all *P* > 0.05), as detailed in Table 1.

**Table 1:** Comparison of clinical baseline data between elderly laparoscopic gastric cancer patients with and without early post-operative enteral nutrition intolerance (*n* = 346).

Variables	Intolerance group (*n* = 194)	Tolerance group (*n* = 152)	*t/χ^2^*	*p*
Age (*y*)	76.85 ± 5.32	71.26 ± 6.18	8.943	< 0.001
BMI (kg/m^2^)	21.35 ± 2.46	22.18 ± 2.31	2.875	0.004
Male/female	108/86	82/70	0.126	0.723
TNM stage (I–II/ III–IV)	65/129	78/74	28.654	< 0.001
Hypertension	85(43.81%)	62(40.79%)	0.428	0.513
Diabetes mellitus	76(39.18%)	35(23.03%)	11.236	0.001
Surgical scope (Limited/Radical)	52/142	68/84	23.567	< 0.001
Operation time (min)	158.62 ± 25.34	132.45 ± 22.18	8.762	< 0.001
Intraoperative blood loss (ml)	285.36 ± 52.47	186.52 ± 45.31	16.893	< 0.001
Anesthesia time (min)	186.45 ± 32.17	162.38 ± 28.56	6.784	< 0.001
Pulmonary infection	32(16.49%)	15(9.87%)	4.215	0.040
Nutrition route (nasal intestinal tube/Nasal gastric tube)	98/96	82/70	1.876	0.171
Enteral nutrition start time (h, post-op)	28.65 ± 6.32	26.38 ± 5.87	3.124	0.002

To explore potential associations between clinical factors and EEN intolerance, Spearman correlation analysis was performed as an exploratory analysis, given the ordinal or categorical nature of several variables. With EEN intolerance status as the dependent variable and all clinical indicators listed in Table 1 as independent variables, the analysis revealed that age, TNM stage, diabetes mellitus, surgical scope, and intraoperative blood loss were positively correlated with EEN intolerance (ρ = 0.685, 0.726, 0.354, 0.658, and 0.762, respectively; all *P* < 0.001). These findings suggested potential associations between these factors and an increased likelihood of EEN intolerance. In contrast, no statistically significant correlations were observed between EEN intolerance and BMI, gender, hypertension, operation time, anesthesia time, pulmonary infection, nutritional support route, or enteral nutrition initiation time (all *P* > 0.05). The detailed results of the correlation analysis are presented in Table 2.

**Table 2:** Spearman correlation analysis between early post-operative enteral nutrition intolerance and potential influencing factors in elderly laparoscopic gastric cancer patients.

Variables	Spearman‘s ρ	*p*
Age (y)	0.685	< 0.001
BMI (kg/m^2^)	−0.125	0.156
Male/female	0.058	0.321
TNM stage	0.726	< 0.001
Hypertension	0.089	0.245
Diabetes mellitus	0.354	< 0.001
Surgical scope	0.658	< 0.001
Operation time (min)	0.186	0.087
Intraoperative blood loss (ml)	0.762	< 0.001
Anesthesia time (min)	0.163	0.112
Pulmonary infection	0.102	0.203
Nutrition route	0.065	0.298
Enteral nutrition start time (h)	0.138	0.135

Correlation coefficients (ρ) represent the strength and direction of associations from Spearman correlation analysis.

All tests were two-tailed, and P < 0.05 was considered statistically significant.

Variables that demonstrated statistically significant differences in univariate analyses (age, BMI, TNM stage, diabetes mellitus, surgical scope, operation time, intraoperative blood loss, anesthesia time, and enteral nutrition initiation time) were entered into a multivariate logistic regression model to identify independent risk factors for early post-operative enteral nutrition (EEN) intolerance. Prior to modeling, multicollinearity among these variables was assessed using the VIF. All VIF values were below 3, indicating no significant multicollinearity and confirming the suitability of the variables for inclusion in the regression analysis.

Multivariate logistic regression analysis revealed that age ≥75 years, TNM stage III–IV, comorbid diabetes mellitus, radical resection, and intraoperative blood loss ≥250 ml were independent risk factors for post-operative EEN intolerance in elderly patients undergoing laparoscopic gastric cancer surgery (all *P* < 0.001). Among these factors, intraoperative blood loss ≥250 ml demonstrated the strongest association with EEN intolerance (odds ratio [OR] = 3.165, 95% confidence interval [CI]: 2.236–4.482), followed by TNM stage III–IV (OR = 2.787, 95% CI: 1.968–3.935). The detailed results of the multivariate analysis, including variable definitions and assignment, are presented in Tables 3, 4.

**Table 3:** Variable definition and assignment for multivariate logistic regression analysis of early post-operative enteral nutrition intolerance in elderly laparoscopic gastric cancer patients.

Factors	Variables	Assignment
Post-operative early enteral nutrition intolerance	*Y*	Yes = 1, No = 0
Age	*X1*	≥75y = 1, < 75y = 0
TNM stage	X2	III–IV (high stage) = 1, I–II (low stage) = 0
Diabetes mellitus	*X3*	Yes = 1, No = 0
Surgical scope	*X4*	Radical resection = 1, Limited resection = 0
Intraoperative blood loss	*X5*	≥250 ml = 1, < 250 ml = 0

**Table 4:** Multivariate logistic regression analysis of risk factors for early post-operative enteral nutrition intolerance in elderly laparoscopic gastric cancer patients.

Variables	β	Wald	OR	95%CI	*p*
Constant term	−2.365	18.963	—	—	< 0.001
Age ≥75y	0.862	26.354	2.369	1.685~3.332	< 0.001
TNM stage III–IV	1.025	38.621	2.787	1.968~3.935	< 0.001
Diabetes mellitus	0.758	21.456	2.135	1.498~3.042	< 0.001
Radical resection	0.986	35.217	2.682	1.895~3.786	< 0.001
Intraoperative blood loss ≥250 ml	1.152	42.857	3.165	2.236~4.482	< 0.001

**Figure 1:**
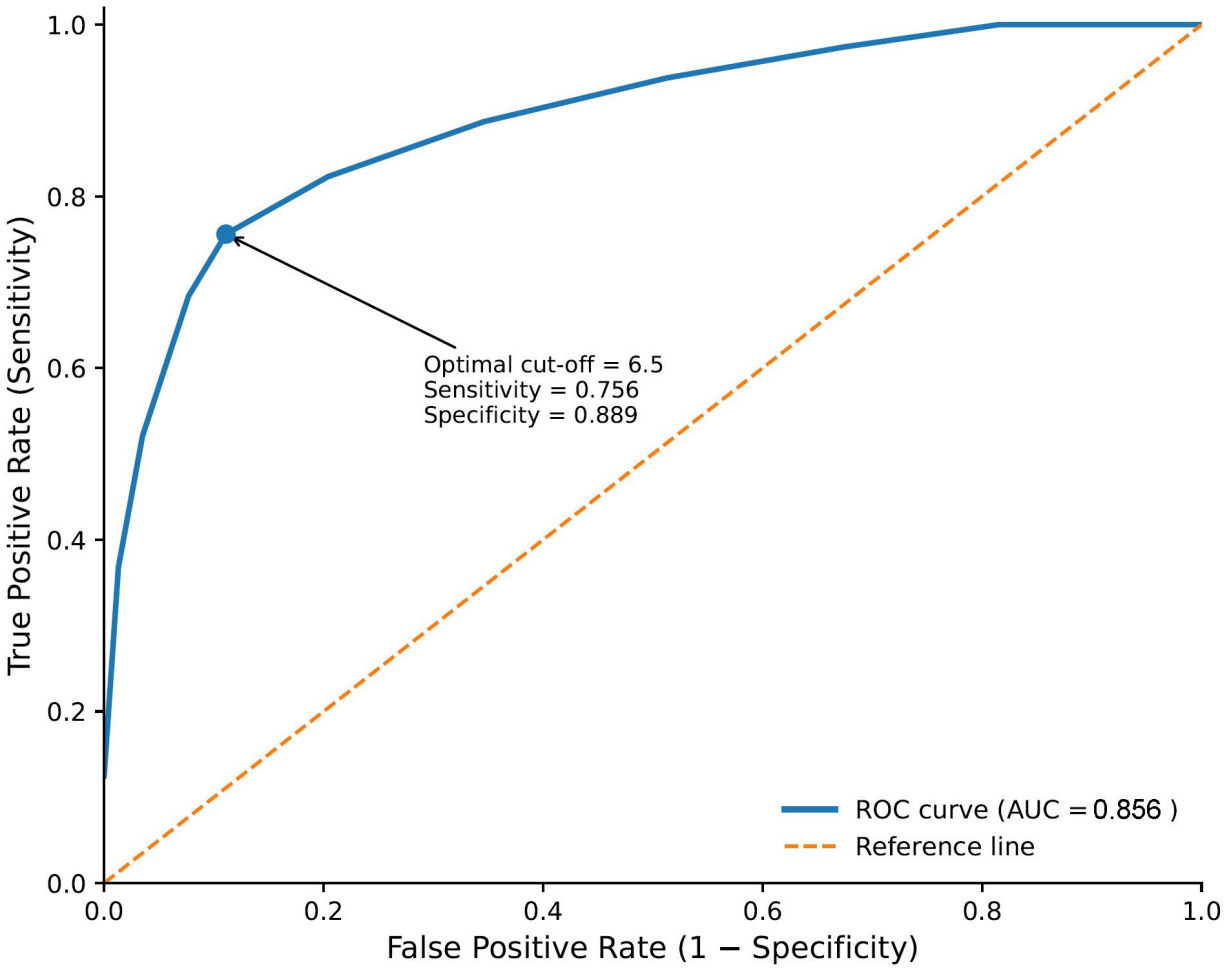
ROC curve for predicting early post-operative en intolerance in elderly laparoscopic gastric cancer patients.

A simplified predictive risk score was constructed based on the five independent risk factors identified above. To enhance clinical applicability and facilitate bedside use, each risk factor was assigned equal weight (2 points), yielding a total score ranging from 0 to 10. The discriminatory ability of the model was evaluated using receiver operating characteristic (ROC) curve analysis (Figure 1). The area under the curve (AUC) was 0.856 (95% CI: 0.812–0.900), indicating good predictive performance.

Analysis of sensitivity, specificity, and Youden index across different cut-off values demonstrated that a risk score of ≥6.5 points provided the optimal threshold for predicting EEN intolerance, with the highest Youden index of 0.645. At this cut-off value, the model achieved a sensitivity of 0.756 and a specificity of 0.889, suggesting favorable predictive accuracy. The complete sensitivity and specificity values at various cut-off points are presented in Table 5.

**Table 5:** Sensitivity, specificity and Youden index of the prediction model for early post-operative enteral nutrition intolerance in elderly laparoscopic gastric cancer patients at different cut-off values.

Total score	Sensitivity	Specificity	Youden index
0.5	1.000	0.000	0.000
1.5	1.000	0.185	0.185
2.5	0.974	0.326	0.300
3.5	0.938	0.487	0.425
4.5	0.887	0.654	0.541
5.5	0.823	0.796	0.619
6.5	0.756	0.889	0.645
7.5	0.684	0.923	0.607
8.5	0.521	0.965	0.486
9.5	0.368	0.987	0.355
10.5	0.125	1.000	0.125

The scoring criteria of the prediction model refer to the 5 risk factors included in Table 4, with 2 points assigned to each risk factor.

The total score ranges from 0 to 10 points (Age ge5year = 2 points, TNM stage III–IV = 2 points, Diabetes mellitus = 2 points, Radical resection = 2 points, Intraoperative blood loss ≥ po ml = 2 points).

The optimal cut-off value is 6.5 points, with the highest Youden Index of 0.645.

## Discussion

This retrospective case-control study analyzed the influencing factors of EEN intolerance in 346 elderly patients undergoing laparoscopic gastric cancer surgery and developed a clinically oriented predictive model. The results demonstrated that the incidence of EEN intolerance was 56.1% in this cohort, with 29.9% of intolerant patients requiring reduction, suspension, or termination of nutritional support. Multivariate analysis identified age ≥75 years, TNM stage III–IV, comorbid diabetes mellitus, radical resection, and intraoperative blood loss ≥250 ml as independent risk factors. These findings are consistent with previous studies (21, 22) on nutritional support in patients, while further refining the risk profile specific to this surgical population.

As a non-modifiable risk factor, the mechanism by which advanced age influences EEN intolerance is well established. Physiological aging is associated with diminished intestinal smooth muscle contractility, slowed peristalsis, and reduced digestive enzyme secretion, all of which impair nutrient digestion and absorption (23, 24). Additionally, elderly patients exhibit blunted stress responses and heightened post-operative inflammatory reactions, which exacerbate gastrointestinal mucosal edema and motility disorders, thereby increasing intolerance risk (25–27). The confirmation of age ≥75 years as an independent risk factor underscores the need for tailored nutritional strategies in older patients.

Tumor stage and surgical factors also play critical roles. Patients with TNM stage III–IV typically present with larger tumor burdens and poorer baseline nutritional status, and often require more extensive surgical resections that cause greater disruption to gastrointestinal anatomy and function (28, 29). Radical resection, compared with limited resection, is associated with longer operative duration, increased surgical trauma, and greater intraoperative blood loss. Significant blood loss may compromise splanchnic perfusion, aggravating mucosal barrier injury (30, 31). Concurrently, surgical trauma-induced activation of the sympathetic-adrenal medullary system inhibits gastrointestinal motility, further pre-disposing patients to intolerance (21).

Diabetes mellitus represents a potentially modifiable risk factor. Chronic hyperglycemia can lead to autonomic neuropathy affecting the gastrointestinal tract, resulting in delayed gastric emptying and dysmotility (32, 33). Post-operatively, diabetic patients exhibit exacerbated insulin resistance and metabolic dysregulation, which may further impair gastrointestinal functional recovery and increase the likelihood of intolerance symptoms such as abdominal distension and diarrhea (34). The higher proportion of diabetic patients in the intolerance group highlights the importance of rigorous perioperative glycemic control.

Based on these findings, we propose a framework for perioperative management. First, risk-stratified intervention: utilizing the predictive model with a cut-off of ≥6.5 points to identify high-risk patients for intensified monitoring and individualized nutritional planning. Second, optimized pre-operative preparation: including strict glycemic control in diabetic patients and pre-operative nutritional assessment with support where indicated (35). Third, refined intraoperative management: minimizing operative time and blood loss, and protecting gastrointestinal mucosal integrity (36). Fourth, precise post-operative nursing: early assessment of gastrointestinal function, gradual initiation of enteral feeding (low concentration and rate), and implementation of interventions such as abdominal massage and early mobilization to promote motility recovery (37, 38). Fifth, dynamic monitoring and adjustment: close surveillance of nutritional status, glycemic levels, and gastrointestinal symptoms, with timely modification of feeding regimens and consideration of prokinetic agents when appropriate (39, 40). It should be emphasized that these recommendations are hypothesis-generating, and their clinical efficacy requires validation through prospective interventional studies. Compared with previously published predictive models for enteral nutrition intolerance (41), our model offers comparable discriminatory ability (AUC 0.856) with the advantage of simplified scoring using equally weighted factors, enhancing its applicability in nursing practice. However, this pragmatic weighting approach, while clinically convenient, may not reflect the differential contribution of each risk factor as indicated by their regression coefficients.

### Limitations and future directions

This study has several limitations that should be considered when interpreting the findings. First, the retrospective, single-center design inherently introduces selection bias and limits the generalizability of the results. The study population was derived from a single institution, and although the sample size was adequate for analysis, multicenter prospective studies are warranted to validate the findings and enhance external validity. Second, the definition of EEN intolerance employed in this study encompassed a broad range of gastrointestinal symptoms (abdominal distension, diarrhea, nausea, vomiting) without consistent documentation of symptom severity, which may have led to overestimation of the true incidence of clinically significant intolerance. While we were able to report that 29.9% of intolerant patients required reduction, suspension, or termination of EEN, detailed severity stratification was not feasible due to the retrospective nature of data collection. Future studies should adopt standardized criteria for grading intolerance severity and document clinical interventions triggered by intolerance symptoms. Third, no internal validation (e.g., bootstrap resampling or split-sample validation) or external validation was performed for the predictive model. Consequently, the reported discriminatory performance (AUC 0.856) may be optimistic, and the model‘s generalizability to other populations remains uncertain. Calibration of the model was also not assessed, which should be addressed in future validation studies. Fourth, although multicollinearity was assessed and found to be acceptable (all VIF < 3), residual confounding from unmeasured variables cannot be excluded. The model did not incorporate potentially important factors such as pre-operative nutritional status (beyond BMI), frailty indices, psychological status, social support, or detailed medication history (e.g., opioids, prokinetics), all of which may influence gastrointestinal function and nutritional tolerance. Fifth, the dichotomization of continuous variables (age ≥75 years, intraoperative blood loss ≥250 ml), while clinically intuitive and based on established thresholds, may have resulted in loss of information and reduced statistical power. This trade-off was accepted to enhance clinical applicability, but future studies with larger samples may consider retaining continuous variables or exploring non-linear relationships. Sixth, the equal weighting of risk factors in the predictive model, while prioritizing simplicity and bedside utility, does not reflect the differential magnitude of effect sizes observed in the regression analysis (e.g., intraoperative blood loss exhibited a stronger association than diabetes mellitus). Alternative weighting approaches based on regression coefficients or odds ratios could yield statistically optimized performance, though potentially at the cost of clinical practicality. Seventh, the observational design precludes any causal inferences regarding the proposed perioperative management strategies. The recommendations outlined in this study should be considered hypothesis-generating rather than evidence-based guidelines.

Future research should prioritize multicenter prospective cohort studies to externally validate the predictive model and assess its performance across diverse populations, incorporating internal validation techniques such as bootstrap resampling and calibration assessment. Standardized definitions and grading systems for EEN intolerance, integrating symptom severity and clinical actions taken, are essential to enable more precise estimation and facilitate cross-study comparisons. Inclusion of additional variables—including comprehensive geriatric assessments, frailty measures, nutritional biomarkers, psychological status, and social support—may enhance model performance and capture the multifactorial nature of intolerance in elderly surgical patients, with machine learning approaches offering potential to identify complex, non-linear relationships among risk factors. Prospective interventional studies, including randomized controlled trials, are warranted to determine whether risk-stratified management based on the model improves clinical outcomes, while long-term follow-up studies should investigate prognostic implications such as nutritional recovery, quality of life, readmission rates, and survival. Health economic evaluations are needed to assess the cost-effectiveness of implementing such strategies in clinical practice. Finally, the development and validation of dynamic prediction models incorporating real-time clinical data may enable adaptive, personalized nutritional management throughout the post-operative course.

## Conclusion

This study finds that post-operative EEN intolerance is common (56.1%) among elderly patients undergoing laparoscopic gastric cancer surgery, with age ≥75 years, TNM stage III–IV, diabetes mellitus, radical resection, and intraoperative blood loss ≥250 ml identified as independent risk factors. The simplified predictive model incorporating these five equally weighted factors (optimal cut-off 6.5 points) exhibited good discriminatory ability in this cohort (AUC 0.856). While the model may serve as a practical tool for risk stratification in nursing practice, its predictive performance requires validation in external cohorts. The proposed perioperative management framework—encompassing risk-stratified intervention, pre-operative optimization, refined intraoperative care, precise post-operative nursing, and dynamic monitoring—represents a hypothesis-generating approach that warrants evaluation through prospective interventional studies. Future research should focus on multicenter external validation, incorporation of additional variables (e.g., psychological factors, frailty), and investigation of the long-term prognostic impact of EEN intolerance in this vulnerable population.

## Data Availability

The original contributions presented in the study are included in the article/supplementary material, further inquiries can be directed to the corresponding authors.

## References

[1] Manceau G Lambert C Pereira B Mantziari S Pasquer A Piessen G . Surgical management of gastric cancer in elderly patients: results of a multicenter cohort of the french surgical association. J Am Coll Surg. (2026) 242:209–22. doi: 10.1097/XCS.0000000000001564 40772696

[2] Chen Y Jia K Xie Y Yuan J Liu D Jiang L . The current landscape of gastric cancer and gastroesophageal junction cancer diagnosis and treatment in China: a comprehensive nationwide cohort analysis. J Hematol Oncol. (2025) 18:42. doi: 10.1186/s13045-025-01698-y 40234884 PMC12001465

[3] Lee HJ Kim YW Park DJ Han SU Ryu KW Kim HH . Laparoscopic pylorus-preserving gastrectomy versus distal gastrectomy for early gastric cancer: a multicenter randomized controlled trial (KLASS-04). Ann Surg. (2025) 281:573–81. doi: 10.1097/SLA.0000000000006503 39219553

[4] Huang ZN Qiu WW Li TY Zhang L She JJ Jia BQ . Comparison of short- and long-term outcomes for robotic versus laparoscopic gastrectomy in elderly patients with gastric cancer: a multicenter cohort study. Surg Endosc. (2025) 39:3860–72. doi: 10.1007/s00464-025-11756-8 40346431

[5] Tian Z Zhang Y Cheng Y Wang D. Long-term muscle-sparing benefits of proximal gastrectomy in elderly patients with upper-third early gastric cancer: a propensity score-matched analysis. Eur J Surg Oncol. (2025) 51:110373. doi: 10.1016/j.ejso.2025.110373 40803191

[6] Urabe M Suzuki M Fukai T Hasegawa Y Terai E Kiya Y . Association of spirometric lung age with survival outcomes in elderly patients undergoing radical surgery for gastric cancer. J Gastrointest Cancer. (2025) 56:225. doi: 10.1007/s12029-025-01355-0 41269479

[7] Wu J Zhang L Ji Y Li H Liu L Zhang X. Effects of enhanced recovery after surgery nursing combined with early enteral nutrition on gastrointestinal function recovery after radical gastrectomy. Langenbecks Arch Surg. (2025) 411:23. doi: 10.1007/s00423-025-03910-6 41307576 PMC12660372

[8] Wang Z Peng W Zhang J. The effect of early enteral nutrition under the ERAS model on gastrointestinal and immune function recovery in patients undergoing gastric tumor surgery. Ann Ital Chir. (2024) 95:1147–54. doi: 10.62713/aic.3738 39723500

[9] Agarwal L Dash NR Pal S Madhusudhan KS Mani V. Single-center randomized trial comparing feeding jejunostomy with nasojejunal tube placement in patients undergoing transhiatal esophagectomy post-neoadjuvant therapy for esophageal cancer. J Gastrointest Cancer. (2024) 55:1282–90. doi: 10.1007/s12029-024-01080-0 38954187

[10] Han L Zhou Y Wang Y Chen H Li W Zhang M . Nutritional status of early oral feeding for gastric cancer patients after laparoscopic total gastrectomy: a retrospective cohort study. Eur J Surg Oncol. (2025) 51:109379. doi: 10.1016/j.ejso.2024.109379 39580263

[11] Cai B Xu G Zhang Z Tao K Wang W. Early oral feeding is safe and comfortable in patients with gastric cancer undergoing radical total gastrectomy. Nutr Cancer. (2025) 77:79–85. doi: 10.1080/01635581.2024.2396150 39188190

[12] Sun S Zheng X Zhang H Han C Zhao G. Hepatic portal venous gas associated with rapid infusion of postoperative early enteral nutrition after radical total gastrectomy. Nutrition. (2022) 101:111685. doi: 10.1016/j.nut.2022.111685 35660505

[13] Aboona MB Wong TW Del Prado PR Paley K Goldberg RF Weimer S . Severe small intestinal bacterial overgrowth syndrome after jejunal feeding requiring surgical intervention: a case report and review of the literature. BMC Gastroenterol. (2022) 22:300. doi: 10.1186/s12876-022-02370-2 35725375 PMC9210687

[14] He FJ Wang MJ Yang K Chen XL Jin T Zhu LL . Nutrients. (2022) 14:17–22. doi: 10.3390/nu14071472 PMC9002901 35406085

[15] Xu Y Hu Q Pei D Zhang Y Zhu H Hui Y . Construction of a preoperative emotional state and postoperative intra-abdominal pressure based prediction model for early enteral feeding intolerance in postoperative patients with gastric cancer. Front Nutr. (2024) 11:1480390. doi: 10.3389/fnut.2024.1480390 39659911 PMC11628306

[16] Zuo L Zhang Z Wang L. Effect of early enteral nutrition after laparoscopic radical gastrectomy on postoperative recovery and inflammatory response. Parenteral Enteral Nutr. (2023) 30:287–91.

[17] Ye P Yuan X Liu Liu C: Research progress on prevention and management of postoperative enteral nutrition intolerance in gastric cancer patients. China J Basic Clin Sur. (2024) 31:1522–7.

[18] Chen J. Construction and verification of a risk prediction model for early enteral nutrition intolerance after gastric cancer resection. Med Theor Prac. (2025) 38:685–8.

[19] Zhao T Le T Baoyin Baoyin S: Risk factors analysis of early postoperative enteral nutrition intolerance in gastric cancer patients under the concept of accelerated rehabilitation surgery Modern Med Health. (2023) 39:3100–4.

[20] Ye P Yuan X Liu C. Research progress on prevention and management of postoperative enteral nutrition intolerance in gastric cancer patients. China J Basic Clin Surg. (2024) 31:1522–7.

[21] Wang S He Y Yi J Sha L. Risk factors for enteral feeding intolerance in critically ill patients: an updated systematic review and meta-analysis. BMC Gastroenterol. (2025) 25:233. doi: 10.1186/s12876-025-03837-8 40200147 PMC11980324

[22] Yuan R Liu L Mi J Li X Yang F Mao S. Early prediction of enteral nutrition feeding intolerance risk in neurocritical patients and development of a simplified risk scoring tables. Eur J Clin Nutr. (2026) 80:159–67. doi: 10.1038/s41430-025-01683-1 41326659 PMC12929056

[23] Yao Y Li H Zhang L. Analysis of high-risk factors for early enteral nutrition intolerance in elderly patients with laparoscopic gastric cancer. J Clin Digest Dis. (2023) 35:344–7.

[24] Lu C. Effect of early enteral nutrition combined with accelerated rehabilitation surgical intervention on improving nutritional status of patients undergoing radical gastrectomy. J Chronic Dis. (2023) 22:1334−7.

[25] Dickerson RN Corley CE Holmes WL Byerly S Filiberto DM Fischer PE. Gastric feeding intolerance in critically ill patients during sustained pharmacologic neuromuscular blockade. Nutr Clin Pract. (2023) 38:350–9. doi: 10.1002/ncp.10911 36156827

[26] McClave SA DiBaise JK Mullin GE Martindale RG. ACG clinical guideline: nutrition therapy in the adult hospitalized patient. Am J Gastroenterol. (2016) 111:315–34. doi: 10.1038/ajg.2016.28 26952578

[27] Lin J Lv C Wu C Zhang H Liu Z Ke L . Incidence and risk factors of nasogastric feeding intolerance in moderately-severe to severe acute pancreatitis. BMC Gastroenterol. (2022) 22:327. doi: 10.1186/s12876-022-02403-w 35780108 PMC9250174

[28] Chen CB. Nutritional and feeding challenges in aerodigestive patients. Curr Opin Pediatr. (2023) 35:561–5. doi: 10.1097/MOP.0000000000001275 37489246

[29] Turner H Morales S Hanson A Carver T. Characterizing gastrostomy tube feeding intolerance in the surgical intensive care unit. J Surg Res. (2025) 317:132–7. doi: 10.1016/j.jss.2025.11.021 41365099

[30] Metheny NA. CE: monitoring adult patients for intolerance to gastric tube feedings. Am J Nurs. (2021) 121:36–43. doi: 10.1097/01.NAJ.0000767356.16777.f1 34255751

[31] Yang H Liu J Sun H. Risk prediction model for adult intolerance to enteral nutrition feeding-a literature review. Am J Med Sci. (2025) 369:427–33. doi: 10.1016/j.amjms.2024.11.012 39617212

[32] Person H Soden J Carey AN Darbari A Khlevner J. Feeding intolerance in adolescents with disorders of gut-brain interaction. J Pediatr Gastroenterol Nutr. (2025) 80:747–50. doi: 10.1002/jpn3.12468 39959957

[33] Kornum DS Krogh K Keller J Malagelada C Drewes AM Brock C. Diabetic gastroenteropathy: a pan-alimentary complication. Diabetologia. (2025) 68:905–19. doi: 10.1007/s00125-025-06365-y 39934370 PMC12021976

[34] Huang X Zhong L Li C Tang Y. Systematic evaluation of risk prediction models for feeding intolerance in ICU patients during enteral nutrition. Asia Pac J Clin Nutr. (2025) 34:577–88. 40738725 10.6133/apjcn.202508_34(4).0009 PMC12504012

[35] Powers J Bourgault AM Carroll Simmons JS. Assessment for enteral feeding intolerance by critical care nurses: a national survey. Dimens Crit Care Nurs. (2025) 44:69–76. doi: 10.1097/DCC.0000000000000685 39853724

[36] Rao Y Chen Y Wang D Chen L Xu X Shen C . Post-pyloric feeding improves the nutritional status of severe tetanus patients and reduces the incidence of feeding intolerance. Clin Nutr ESPEN. (2025) 68:509–14. doi: 10.1016/j.clnesp.2025.05.047 40480462

[37] Liu L Li J Hu L Cai X Li X Bai Y. Development and validation of a prediction model for enteral feeding intolerance in critical ill patients: a retrospective cohort study. J Clin Nurs. (2025) 34:2336–47. doi: 10.1111/jocn.17660 39888094

[38] Panwar R Kumar N Parikh H Dash S Rai S. Standard continuous feeding versus intermittent feeding among mechanically ventilated patients in intensive care: a systematic review and meta-analysis of randomized controlled trials. Clin Nutr. (2025) 51:40–9. doi: 10.1016/j.clnu.2025.05.024 40516326

[39] Mohajeri L Daghayeghi R Rostami N Moeinipour Y Zadeh RH Zadeh RH . Early oral feeding after laparoscopic total gastrectomy in gastric cancer patients: a meta-analysis of randomized controlled trials and cohort studies. BMC Gastroenterol. (2025) 25:709. doi: 10.1186/s12876-025-04283-2 41068719 PMC12512641

[40] Wang Y Li Y Chen Y Zhang Y Feng X Lv D . Modifying feeding protocols in critically ill patients based on a predictive model of feeding intolerance: protocol for a multicenter cluster randomized controlled trial (the mNEED study). Front Med (Lausanne). (2025) 12:1649983. doi: 10.3389/fmed.2025.1649983 41281994 PMC12630114

[41] Wang G Lu C Solomon OM Gu Y Ling Y Xu F . Construction and evaluation of a machine learning-based predictive model for enteral nutrition feeding intolerance risk in ICU patients. Front Nutr. (2025) 12:1600319. doi: 10.3389/fnut.2025.1600319 40704315 PMC12283280

